# Controlling simonkolleite crystallisation *via* metallic Zn oxidation in a betaine hydrochloride solution

**DOI:** 10.1039/d3na00108c

**Published:** 2023-03-02

**Authors:** Shaoqing Qu, Eftychios Hadjittofis, Francisco Malaret, Jason Hallett, Rachel Smith, Kyra Sedransk Campbell

**Affiliations:** a The University of Sheffield, Department of Chemical and Biological Engineering Sheffield UK k.sedransk@sheffield.ac.uk; b UCB Pharma SA Belgium Brussels Belgium; c Imperial College London, Department of Chemical Engineering London UK; d Nanomox Ltd. London UK

## Abstract

Zinc oxide nanoparticles, with a hexagonal flake structure, are of significant interest across a range of applications including photocatalysis and biomedicine. Simonkolleite (Zn_5_(OH)_8_Cl_2_·H_2_O), a layered double hydroxide, is a precursor for ZnO. Most simonkolleite synthesis routes require precise pH adjustment of Zn-containing salts in alkaline solution, and still produce some undesired morphologies along with the hexagonal one. Additionally, liquid-phase synthesis routes, based on conventional solvents, are environmentally burdensome. Herein aqueous ionic liquid, betaine hydrochloride (betaine·HCl), solutions are used to directly oxidise metallic Zn, producing pure simonkolleite nano/microcrystals (X-ray diffraction analysis, thermogravimetric analysis). Imaging (scanning electron microscopy) showed regular and uniform hexagonal simonkolleite flakes. Morphological control, as a function of reaction conditions (betaine·HCl concentration, reaction time, and reaction temperature), was achieved. Different growth mechanisms were observed as a function of the concentration of betaine·HCl solution, both traditional classical growth of individual crystals and non-traditional growth patterns; the latter included examples of Ostwald ripening and oriented attachment. After calcination, simonkolleite's transformation into ZnO retains its hexagonal skeleton; this produces a nano/micro-ZnO with a relatively uniform shape and size through a convenient reaction route.

## Abstract 2

Zinc oxide nanoparticles, with a hexagonal flake structure, are of significant interest across a range of applications including photocatalysis and biomedicine.

## Graphical abstract

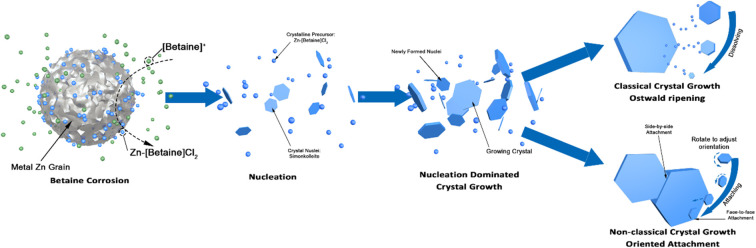

## Introduction

The use of zinc oxide (ZnO) as an additive crosses a diverse range of applications, including skincare,^1^ photocatalysis,^2^ electronics,^3^ and biomedicine.^4^ Some applications require tightly controlled ZnO, whilst others do not currently have the same high specification requirements. The use of highly-specified ZnO is for applications where specific functionalities of ZnO^5^ are achieved, these function are linked to particular structural features (including at the nanoscale). Thus, the development of ZnO micro and nanomaterials has been of significant interest;^6^ more applications may benefit as the both the structure–property relationships are better understood and the ability to achieve these structures is improved. A wide breadth of ZnO morphologies have been reported, using various synthetic approaches, including rod,^7^ spherical,^8^ needle-like,^9^ nanodisk,^10^ and more complex assembled structures.^11,12^ Some of the synthetic routes look to intermediates to produce additional morphologies. Li^13^ reported nanosheets of ZnO formed using simonkolleite as an intermediate; this pathway could serve to synthesise (thin) hexagonal nano and micro disks, on a large scale.

**Fig. 1:**
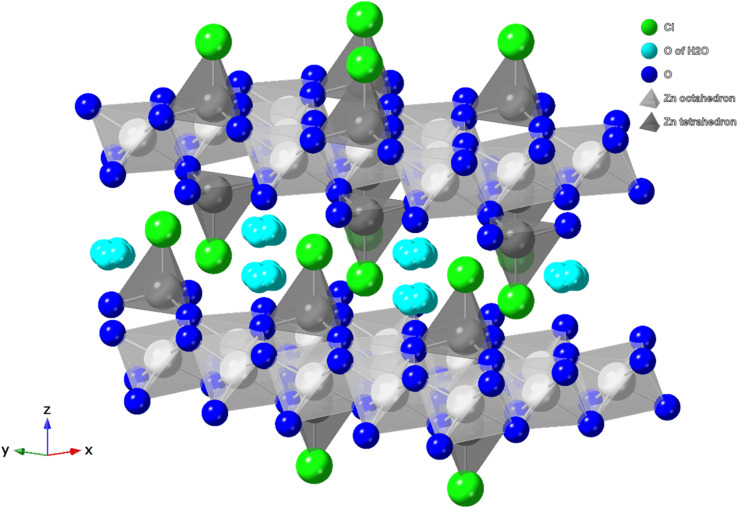
The layered double hydroxide crystal structure of simonkolleite.^15,60^ Zinc containing octahedron and tetrahedrons are shown (light and dark grey, respectively) with the interlayer spacing containing water. N. b. H atoms are not shown.

Simonkolleite (Zn_5_(OH)_8_Cl_2_·H_2_O) is a layered double hydroxide (LDH) which was first reported by Schmetzer *et al.*^14^ (Fig. 1). The crystal comprises a stack, along the *c*-axis, of flat layers; these layers are composed of octahedron oxyhydrogen complexes centered on zinc (light grey) and tetrahedrons of the ZnO_3_Cl complex (dark grey). The structure is completed with water molecules between layers^15,16^ and Cl atoms preferentially directed into the same interlayer space.

Interest in simonkolleite itself stems from several desirable properties. The layered structure has excellent potential as a cation exchange material,^17^ is popular as a filler for nanocomposites,^18^ has been employed as a drug carrier in systems requiring slow-release,^19–21^ and is appealing as a catalyst or sieve.^22^

Synthesis of simonkolleite has been reported, including by Li,^13^*via* precipitation in an ammonia solution, using ZnCl_2_ as the zinc source. Subsequent calcination of the simonkolleite produced both sheet-like and spindle-like ZnO structures, with a thickness of about 40 nm. Nanosheet ZnO has been confirmed to have good activity as a photocatalyst. However, this process lacks key environmental and sustainability credentials, which severely detracts from its potential in future deployment; the concerns include: (1) the volatility and corrosiveness of ammonia, (2) the precise pH control reportedly required, and (3) the unsustainable and environmentally-damaging waste stream produced. To improve the sustainability of this method, Aida^23^ reports the use of hibiscus flower extract. However, this change is incremental and does not address the high concentration of ZnCl_2_ required as a reactant. Other conventional synthetic methods, such as using sodium hydroxide to carry out precipitation, neither provide good morphological control of the products nor a greener chemistry.^24–26^

**Fig. 2:**
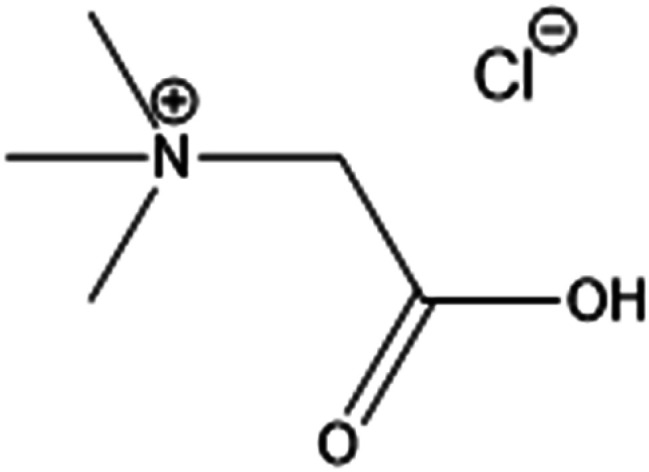
The molecular structure of betaine hydrochloride (betaine·HCl).

Betaine hydrochloride (betaine·HCl) is in the family of choline carboxylate ionic liquids (Fig. 2). It is composed of a hydrophilic carboxylic tail with a hydrophobic quaternary ammonium salt head. Its amphoteric structure leads to specific aggregation behaviours in the liquid phase, distinguishing it from conventional solvents or salts. It has been suggested that the hydrogen bonding of betaine·HCl cation and water molecules can serve as a mechanism to link two or more molecules; this then serves as a basis for the formation of aggregates.^27,28^ The self-assembly behaviour of betaine·HCl molecules may affect crystal growth; thus, it has the potential to be used to adjust, and possibly control, crystal structure.^29^ Furthermore, betaine·HCl can be synthesised from biomass in a sustainable and economical way.^30^ Compared with conventional solvents or other ionic liquids, such as imidazolium-based ionic liquids, betaine·HCl has lower toxicity and is easily degraded without significant environmental impact.^31–33^ It has been employed in the metal industry, *e*.*g*. rare-earth metal recovery^34^ and metal complex synthesis.^35,36^ Our previous work have demonstrated betaine·HCl can dissolve Zn, and has been reported to undergo complexation.^37,38^ The implications, therefore, include the possibility of inducing the precipitation of simonkolleite with a specific nano/micro structure.^39^

Currently, to the best of our knowledge, simonkolleite synthesis routes employing conventional solvents rely on precise pH adjustment to prevent competing reactions (in turn, resulting in the formation of other zinc complexes like Zn(OH)_2_).^18^ In previous work, an alternative method was developed to produce simonkolleite from zinc using aqueous solutions of 1-butyl-3-methyl-imidazolium chloride.^40^ Ionic liquid 1-butyl-3-methyl-imidazolium chloride is employed to catalytically oxidise metal Zn. However, the reaction pathway to simonkolleite is both slow and goes through multiple intermediate species. The consequence of this is both a variety of products (including Zn(OH)_2_, for example) and the yield of simonkolleite is relatively low. It exposed that conventional ionic solutions is difficult to control the morphology of the zinc product, by virtue of the solvent alone without adding additional reagents.

Betaine·HCl can oxidise metal zinc directly, offering an alternative to 1-butyl-3-methyl-imidazolium chloride, inducing a high concentration of Zn^2+^ ions. However, its differences from 1-butyl-3-methyl-imidazolium chloride present an opportunity to reduce, and potentially all-together avoid, the formation of most competing products or the presence of polymorphism. Meanwhile, it is also different from conventional acids like HCl to form hydrogen and stop at ZnCl_2_.^41^ Without manual pH adjusting, Zn in betaine hydrochloride solution, can stably produce high-purity, regular hexagonal sheet-like simonkolleite, which outperforms conventional acids. Thus, this economical green ionic liquid, betaine·HCl, presents excellent potential in synthesising simonkolleite nano/microparticles, which can be subsequently calcined into ZnO nanosheets. Meanwhile, the expected waste stream is deacidified betaine, which is easy to be reused by acidification.^42,43^

Herein, this work is based on the utilisation of recycled scrap metal Zn to synthesise shape-controllable simonkolleite crystals. The conventional acid and additives are replaced by betaine hydrochloride, as an environmentally-benign solvent to produce simonkolleite through the direct oxidation of a solid Zn metal substrate. Adopting mild and environmentally friendly betaine acid minimised the potential risk to operators and the environment. Simultaneously, this process also eliminates the need to adjust the pH of the solution. It developed a ‘one-pot’ method, which simplified the complexity of the operation as much as possible, to achieve precise regulation of the morphology of simonkolleite. Whilst there are sustainability benefits to removing this salt, in fact, its addition through the solid metal is grounded in more fundamental reasoning. The slow release of zinc ions into the liquid phase is achieved by the moderate reaction rate of betaine·HCl with zinc. The zinc concentration in the liquid phase is essentially being managed, with a view towards maintaining a favourable concentration range for the formation of well-controlled simonkolleite crystals. This ingenious solvent utilisation method has potential in the crystallisation industry. The products were characterised by X-ray diffraction (XRD), thermogravimetric analysis (TGA), and scanning electron microscopy (SEM) to verify the purity of the product simonkolleite and the feasibility of converting it into flaky ZnO. Using this pathway, relatively pure simonkolleite with flaky hexagonal nanocrystals can be obtained. This study mainly investigated the morphological effects of betaine·HCl concentration, reaction time, and reaction temperature on the simonkolleite formed. Additionally, it also obtained ZnO nanoparticles by calcination experiments to verify the feasibility of producing flaky hexagonal ZnO.

## Experimental methods

This experiment used metal zinc (Zn grains, 20–35 mesh, 99.8%, Sigma-Aldrich) as the Zn source. Other reagents included betaine·HCl (betaine hydrochloride, PCR reagent, >99.9%, Sigma-Aldrich), methanol (anhydrous, 9, 9.8%, Sigma-Aldrich) and deionised water. All reagents were used as supplied (*i*.*e*. without any further purification or treatment).

### Synthesis of simonkolleite

#### Room temperature leaching synthesis

The matrix of conditions used in the synthesis of simonkolleite are in Table 1. The concentration of the aqueous betaine·HCl solution was selected as 10 and 40% of the mass fraction (2.8 and 10.5% mol fraction); the former is referred to as ‘high’ water case and the latter is referred to as the ‘low’ water case. For both solution concentrations, reactions were run for 1, 3, 5, 7, and 15 days. In each case, the 15 mL of betaine·HCl solution was mixed with 3 g of Zn grains in a flask. The flasks were placed in a shaking incubator (SciQuip, Incu-Shake series) at 200 rpm and 40 °C.

**Table 1:** Simonkolleite synthetic ingredients list

Serial number	Betaine·HCl concentration/mass%	Mass of solution/g	Mass of Zn grains/g	Reaction temperature/°C	Reaction time/days
1	10	15	3	40	1
2					3
3					5
4					7
5					15
6	40				1
7					3
8					5
9					7
10					15
11	40	10	2	160	1

#### High-temperature hydrothermal synthesis

A complementary experiment was studied at an elevated temperature of 160 °C for 24 hours to observe the relative formation of simonkolleite. The conditions (noted in Table 1) were at the ‘low’ water concentration (*i*.*e*. 40% mass fraction of betaine·HCl) with 2 g of Zn grains and 10 mL solution in a 50 mL autoclave.

#### Separation, washing and drying of products

**Fig. 3:**
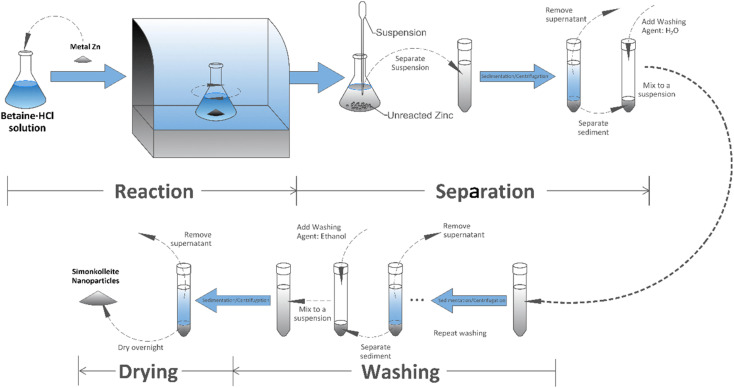
Experimental process flow schematic diagram.

After the reaction had reached the specified time, the mixture was stirred sufficiently such that the powder remained suspended in the liquid phase. The product was separated by a combination of settling and washing steps (Fig. 3). The suspension was separated from the solid phase into a falcon tube, unreacted zinc grains remained at the bottom of the reactor. The suspension contained a large quantity of white precipitate at the bottom of the tube by standing overnight. The supernatant was removed by pipette manually. The residual solid sample was added to 10 mL of deionised water to prepare for further washing. Centrifugation (VWR Mega Star 3.0) was employed at 1000 rpm for 3 minutes, where the parameters were selected based on the properties of both the simonkolleite and solution. These steps were repeated until no solid product was retrieved, and a final methanol wash step was used. The precipitate was dried in the fume hood and weighed once dry.

#### Characterisation

The composition and crystal structure of the products were characterised by X-ray Diffraction (XRD, Bruker D2 Phaser, 30 kV, 16 mA). The product morphologies were observed by Scanning Electron Microscopy (SEM, JSM-6010LA InTouchScope, 20 kV). Due to the hexagonal shape, and packing, of the particles, the side lengths of hexagons were used to quantify particle size. Analysis included regularity of hexagons; large sample sizes were used (*n* = 200) to ensure good representation of the population. Herein, the particle size refers to the side length of a single hexagon unless stated otherwise.

The products also were characterised by thermogravimetric analysis (TGA 4000, PerkinElmer, Inc.). For each sample, *ca*. 15 mg was transferred into a crucible. A N_2_ gas environment with a flow rate of 20 mL min^−1^ was used. The sample was heated from 30 °C to 800 °C at a rate of 1 °C min^−1^ and held for 30 minutes. Subsequently, the sample, now calcined, was cooled to room temperature at 5 °C min^−1^.

## Results and discussion

Three variables involved in the synthesis process of simonkolleite were considered: betaine·HCl concentration, reaction time, and reaction temperature. Two betaine·HCl concentrations were probed (‘low’ and ‘high’ water content) as a function of time (up to 15 days) at 40 °C; a separate case investigated the effect of elevated temperature (160 °C).

To illustrate key features of the product characterisation, a case study (10% betaine·HCl solution for ten days) is presented in detail below. Subsequently, a discussion of the morphological control of simonkolleite particles (size and thickness), as a function of varying reaction conditions (betaine·HCl concentration, reaction time, and reaction temperature) is presented.

### Product characterisation

The product from 10% of betaine·HCl after a 10 day reaction period is used as the exemplar herein to illustrate the characterisation undertaken. As previously described, the solid product was recovered through washing steps; subsequent analysis was executed to obtain information on composition and morphology of the solid particles using XRD, SEM, and TGA.

**Fig. 4:**
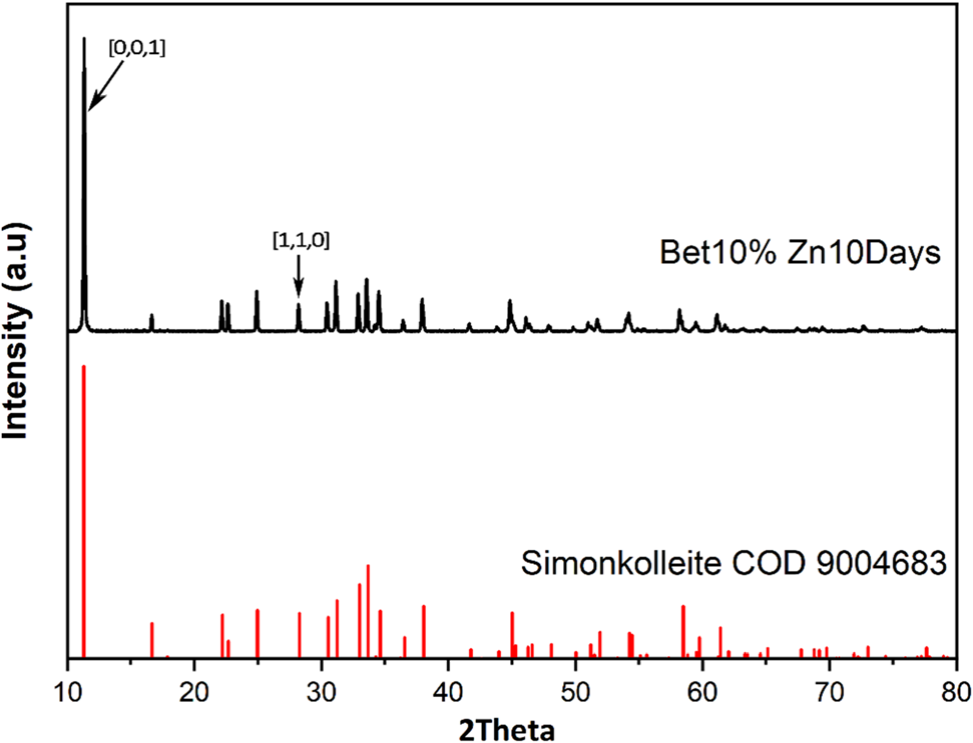
The XRD patterns of the JCPDS standard peak of simonkolleite (COD 9004683) (bottom diffraction pattern shown in red) and the synthesised product recovered from 10% of betaine·HCl after a 10 day reaction period (top diffraction pattern shown in black).

For the separated product, a white powder, has XRD peaks strongly consistent with simonkolleite (Zn_5_(OH)_8_Cl_2_·H_2_O) (Fig. 4), suggesting the synthesised product has relatively high purity. The product has a strong peak where 2*θ* is *ca*. 11°, corresponding to the [0,0,1] crystal plane of simonkolleite.^13,44^ The intensity of the peak attributed to the [0,0,1] plane implies that the end-face is, by far, the dominant face exposed by the crystal. Moreover, it indicates that the growth along the *c*-axis is inhibited. Taken together this describes the crystals presenting as flat, rather than elongated rods.

**Fig. 5:**
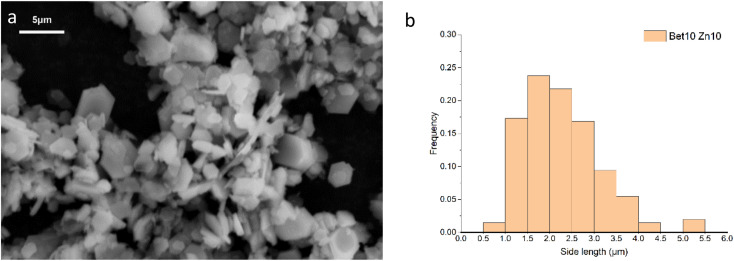
(a) SEM images of product simonkolleite; (b) particles size distribution for the case of 10% of betaine·HCl with a 10 day reaction. (b) The sample presented is for *n* = 200, where hexagonal crystals showed regularity (*i*.*e*. regular hexagons). The distribution step-size selected is 0.5 μm, based on measurement confidence.

The synthesised simonkolleite was imaged (Fig. 5a) using SEM. These images are consistent and regular hexagonal flake morphology is observed, which is consistent with the typical crystal form of simonkolleite and in good agreement with the analysis of the XRD diffraction pattern obtained. The clean, sharp edges of the crystals are notable and provide strong evidence that product is fully crystalline; this is also consistent with XRD where peaks show good definition. Neither technique gives indication of amorphous material.

The length of one edge of an individual hexagonally shaped crystal was selected as a metric to quantify particle size. The regularity of hexagon side lengths was assessed and found that the edge length variance of three sides for one crystal was, on average for *n* = 200, <0.01 μm, the effective limit of measurement accuracy and confidence. As such, hexagons measured could be considered regular hexagons within the measurement limit. The distribution of side lengths for *n* = 200 was in the range between 0.5 μm and 5.5 μm. Due to the clustering of crystals, the thickness cannot be measured reliably; one can only assuredly state that the thickness of a single crystal is <0.5 μm. The presence of clustering and/or sticking will be discussed in later sections. The product characterisation by thermogravimetric analysis (TGA), consistent with previously reported studies for simonkolleite,^45^ is discussed in a later section.

### Effects of reaction conditions on the morphology

Crystal morphologies are often closely linked to functional properties, providing significant importance in understanding how specific morphologies are achieved. The crystal morphologies of simonkolleite are influenced by the synthesis conditions, including betaine·HCl concentration, reaction temperature, and reaction time.

#### Betaine·HCl concentration

**Fig. 6:**
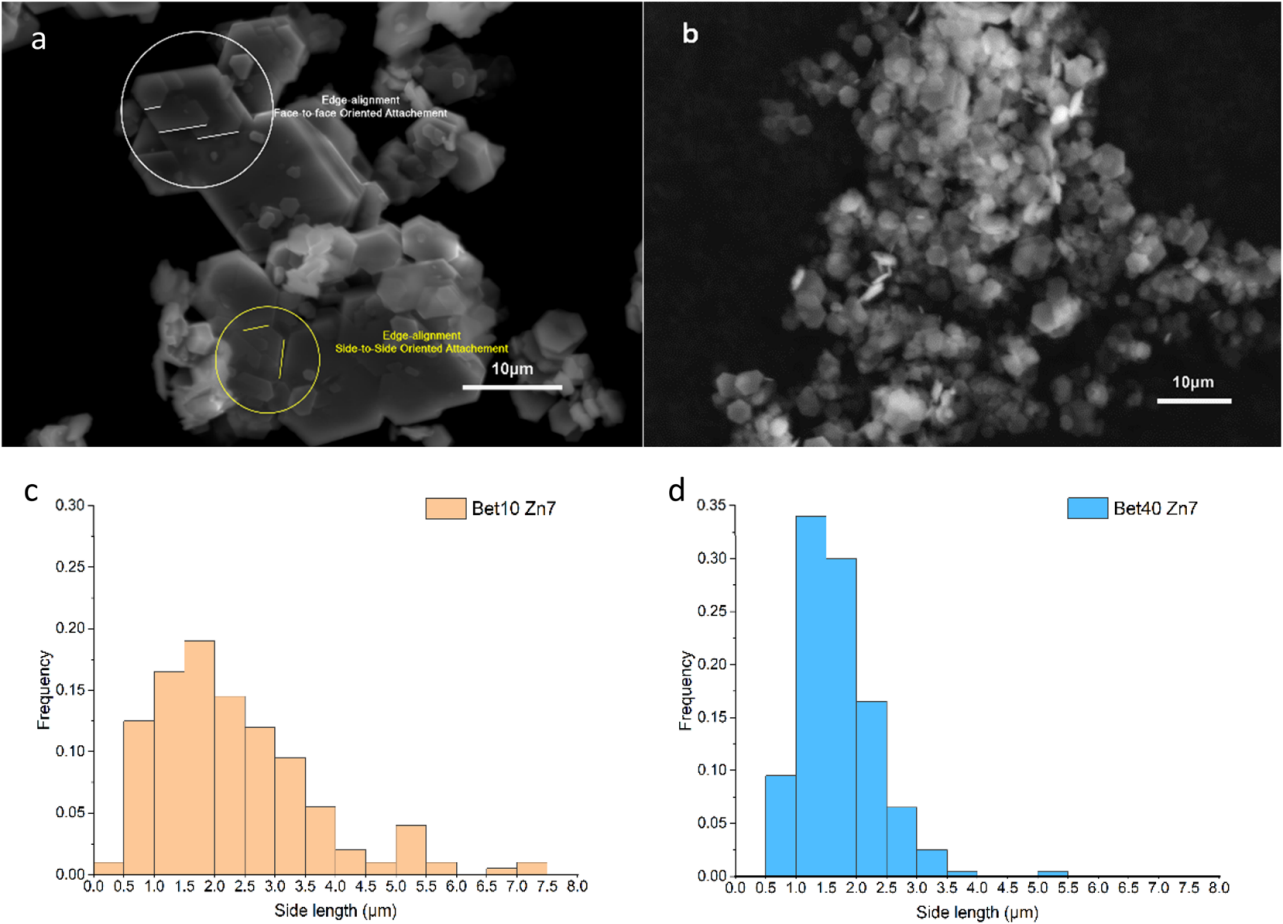
SEM images (a and b) and their corresponded size distribution maps (c and d) of simonkolleite synthesized in the betaine solution for seven days with different concentration. Images, using SEM, are shown for (a) simonkolleite synthesized in 10% betaine·HCl. The crystal structure inside the circles show two different types of oriented attachment; (b) simonkolleite synthesized in 40% of betaine·HCl. The size distribution maps are included below the respective SEM image where the length represents a side length of the regular hexagons for *n* = 200 for (c) 10% of betaine·HCl and (d) 40% of betaine·HCl.

Regardless of the betaine·HCl solution concentration (10 *versus* 40%), hexagonally shaped crystals were formed. However, significant distinctions in the size was observed when comparing those synthesised in 10% of betaine·HCl, as compared to 40%, both after seven days (Fig. 6). In the 10% betaine·HCl, the distribution has a mean of *ca*. 2.3 μm and median of *ca*. 2.0 μm; 90% of the crystals measured fall in the range of 1.0 to 4.0 μm with a long tail making up the remainder (Fig. 6c). As previously noted, quantification of the crystal thickness is subject to inaccuracies. However, qualitatively it is apparent that larger hexagonal crystals (side length *ca*. 4.0 μm) appear to have thicknesses reaching 2 μm. But the majority of small crystals (side length *ca*. 2.0 μm) appear to have a thickness of <0.5 μm.

**Fig. 7:**
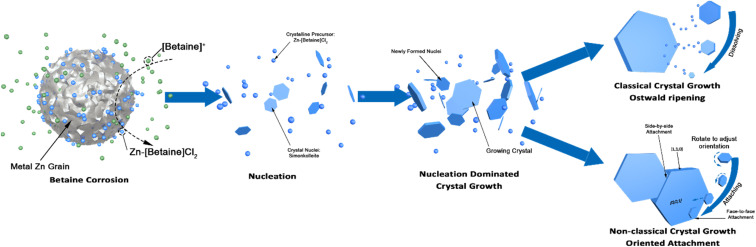
Schematic diagram of the crystallisation process of simonkolleite in betaine·HCl solution. In the first step, free betaine ions (green balls) react with the metallic Zn surface, which can then go on to form Zn-[betaine]Cl_2_ complexes (blue balls). This reaction, from the free to coordinated betaine ions, has a likely intermediary step where Zn ions accumulate in the liquid phase. The Zn-[betaine]Cl_2_, which is the precursor of simonkolleite, increases in concentration to supersaturation levels and the hexagonal nucleus starts to form. When the Zn-[betaine]Cl_2_ concentration is still high, nucleation-dominated growth will be prevalent. Subsequently, due to consumption of the precursor the supersaturation drops and the primary mechanism shifts away from nucleation-dominated to a combination of Ostwald ripening and oriented attachment.

The wide distribution in the 10% betaine·HCl case can be attributed to two different non-classical crystal growth mechanisms: Ostwald ripening^46^ and oriented attachment (Fig. 7). Initially in the solution, the interaction between metal zinc and betaine·HCl results in the release of crystalline precursors, which are most likely Zn-[betaine]Cl_2_.^47,48^ In the early growth stages, the simonkolleite crystals then grow following the classical growth mechanism, from crystalline precursors, into tiny (and growing) crystals.^49^ However, not all crystal precursors will follow this pathway. When the level of supersaturation decreases, non-classical crystallisation mechanisms will overtake the classical mechanism. This shift will cause tiny crystals to support simonkolleite growth through a sacrificial mechanism, Ostwald ripening. The tiny crystals dissolve, and their dissolution then supports the further growth of other pre-existing (larger) crystals.^50^

In addition to growth through the Ostwald ripening process, there is also evidence of growth by oriented attachment. Through imaging two observations are made which support the existence of this second mechanism: (1) large-scale aggregation amongst individual crystals and (2) multiple tiny crystals oriented and attached to large crystal surfaces. In particular, a common approach to oriented attachment can be described as face-to-face attaching; the [0,0,1] planes are stacked on each other, resulting in the tiny single crystals merging into thicker large crystals after alignment. Another type of attachment, side-to-side, is also observed; large crystals undergo ordered fusion in the thin side plane. Through these side-to-side connections, multiple hexagons can be aggregated into complex flat clusters at higher scales.^51^

The oriented attachment is a non-classical mechanism impacting the crystal coalescence process.^52^ Multiple crystals are induced by the anisotropy of each plane or interaction, such as face-specific van der Waals forces and electrostatic interactions, adjusting the contact angle between crystals to minimise the surface energy.^53^ Whether it is the attachment of tiny crystals or the fusion of large crystals, the alignment is preferential over disorder. Therefore, it can be deduced that the crystal formation of simonkolleite in 10% of betaine·HCl is affected by the combined action of Ostwald ripening and oriented attachment.

By contrast to the simonkolleite formed at 10%, that which was precipitated from a 40% betaine·HCl solution exhibits a more uniform and smaller size distribution. The crystals are easily identifiable individually and have a single side particle length mean of *ca*. 1.7 μm and median of *ca*. 1.6 μm (Fig. 6b and d). The crystals made at these ‘low’ water content conditions have a significantly thinner appearance (so much so that they appear nearly translucent under the SEM). Evidence of growth patterns here are different, with indications pointing to nucleation-dominated crystallisation (not growth-dominant crystallisation). Notably, there is also no observed directional attachment or coalescence.

#### Reaction time

In the previous section, focusing on concentration of betaine·HCl in solution, the time step of seven days was used to illustrate a significant difference between particles generated at 10 *versus* 40% betaine·HCl solutions. These distinctions point to the existence of different mechanisms in play. In this section, the previously proposed mechanisms are probed, and further explored, as a function of time.

**Fig. 8:**
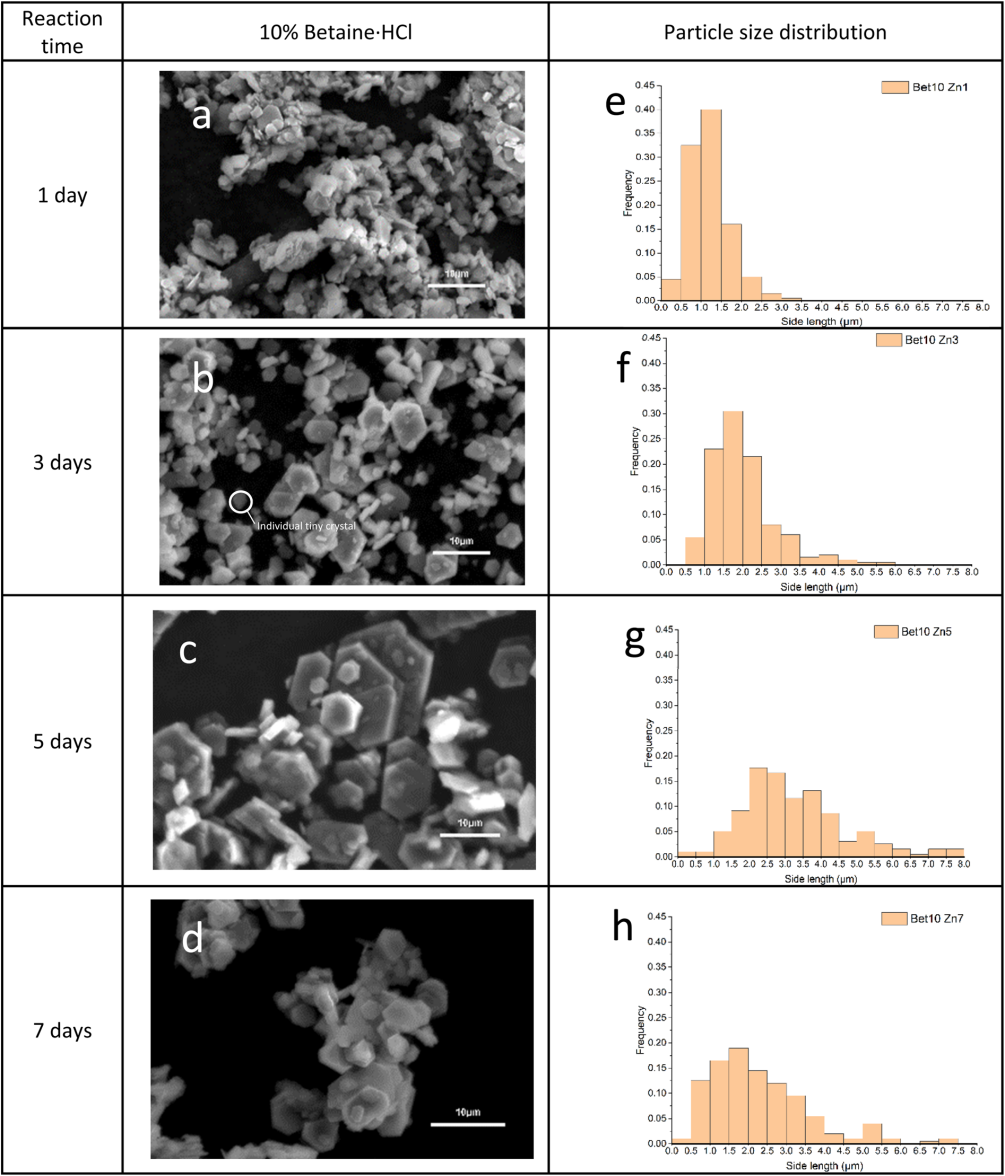
SEM images and their corresponded size distribution charts of simonkolleite (*n* = 200 for each experiment) are shown for (a and e) one, (b and f) three, (c and g) five, and (d and h) seven days in 10% betaine·HCl.

Simonkolleite single-crystals formed in 10% betaine·HCl solutions maintain a hexagonal shape, as a function of time, during the crystallisation growth process (*i*.*e*. no observation of any significant transition or polymorphism) (Fig. 8). With increasing time, the hexagonal crystals show an increase in size of both the face and the plate thickness. After one day in 10% betaine·HCl solution (Fig. 8a), the crystals appear as separate hexagonal sheets, suggesting a classical nucleation and growth sequence of mechanisms. The crystal flakes' mean side length was *ca*. 1.3 μm (Fig. 8e). The thickness of the flakes observed was still relatively thin. With increasing time, two significant changes occurred. Initially, the primary individual crystals increased in size in the [1,0,0] and [1,1,0] directions. A shift in the average side length of the hexagons is evidenced across the period from one to seven days. At seven days, the side length reached a mean of *ca*. 2.3 μm. However, the increasing average is only one metric; the particle distribution is gradually widening with time. This latter phenomenon is unsurprising as various merging and aggregation behaviours are clearly occurring (Fig. 8b–d), where the various pathways differentially alter the crystal growth, and therefore size (Fig. 8f–h).

During this process, imaging suggests that the number of tiny crystals gradually declined with increasing time. It can be observed that some individual tiny crystals were still present in the third-day sample, while they were rarely found in the seventh-day sample. This pathway illustrates the previously discussed process of nucleation and growth. In the early phase, tiny crystals may grow; however, with increasing time, the majority of the tiny crystal population either have grown themselves or have been incorporated into larger-diameter structures by Ostwald ripening or oriented attachment.

Therefore, single crystals rarely grow individually and independently to a diameter >5 μm. This is evidenced by the fact that crystals of this size no longer appear to be from a single nucleus, and notably the hexagonal shape is maintained regardless of multiple size crystals coordinating. Considered all together, this continues to provide substantial evidence of both Ostwald ripening^54^ and oriented attachment^55,56^ occurring in the crystallisation process, furthermore this then also indicates the self-assembly capability of simonkolleite.

**Fig. 9:**
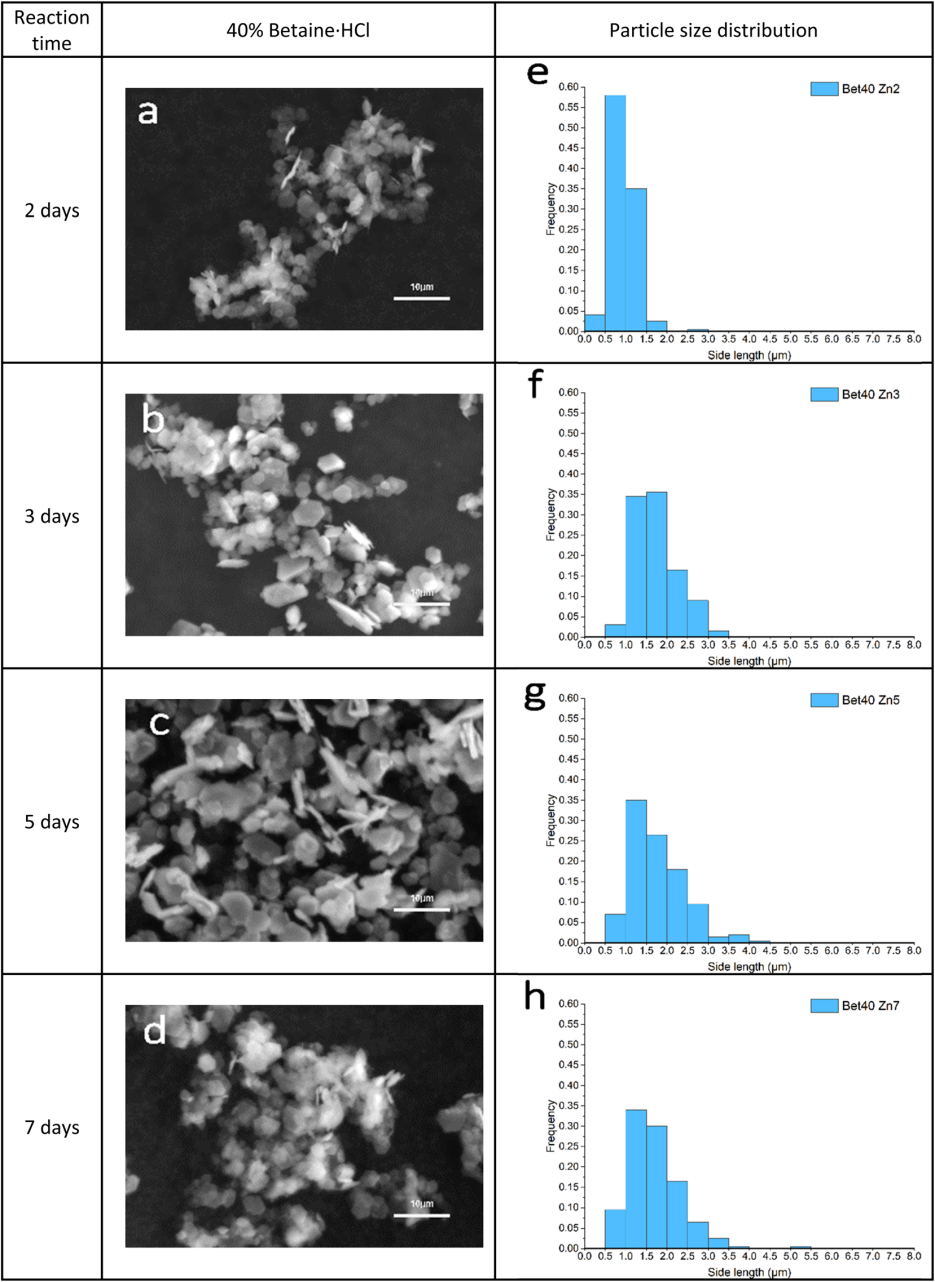
(a–d) SEM images of simonkolleite crystal growth synthesised in 40% of betaine·HCl over time; (e–h) corresponded PSD maps statistical the side lengths of 200 hexagonal crystals.

Precipitation of crystal product in the 40% of betaine·HCl was not observed, nor could any product be precipitated, after one day. The first solid precipitation was separated successfully after two days (Fig. 9a). Although the product in 40% concentration remains hexagonal shape, the crystal growth process is different from the 10% case. The particle size increased with increasing reaction time; however, the crystal size distribution is not broadening with time as was seen in the 10% case. Individual crystals have a mean side length of *ca*. 1.5 μm; no crystals' side lengths were ever measured to be >4 μm (Fig. 9e–h). The crystals' thickness can only be assessed semi-quantitatively, due to the nature of the sample and imaging; however, there is virtually no evidence of increasingly thick crystals, as is seen in the 10% case. The crystals do not exhibit attachment mechanisms (of either the face or side), leaving most as individual crystals without agglomeration. The relatively narrow size distribution also suggests that Ostwald ripening is either uncommon or does not occur at all.

**Fig. 10:**
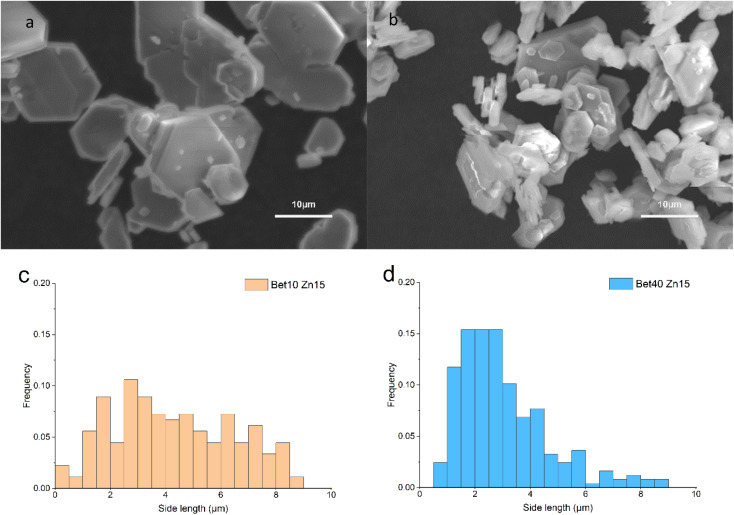
SEM images (a and b) for simonkolleite synthesized, after 15 days, and the size distribution of the side lengths (*n* = 200 hexagonal crystals) (c and d) for 10% of betaine (a and c) and 40% of betaine (b and d).

Whilst distinctions were obvious in the samples assessed at seven days, a qualitative look at a longer reaction time of 15 days (Fig. 10) shows fewer notable differences. In both cases, there are several key features observed: (1) some crystals are >10 μm in diameter and (2) there is evidence of attachment and/or merging. What does remain distinguishable, however, is that the particle size distributions are consistent with what was observed at earlier time steps. For 40% betaine·HCl the distribution is narrower, with a *ca*. 90% of the crystals between 0 and 5 μm for side length. By contrast, the 10% betaine·HCl is much broader in its distribution and with no real peak; to provide a comparison, the proportion of crystals between 0 and 5 μm is only 61.5%.

**Fig. 11:**
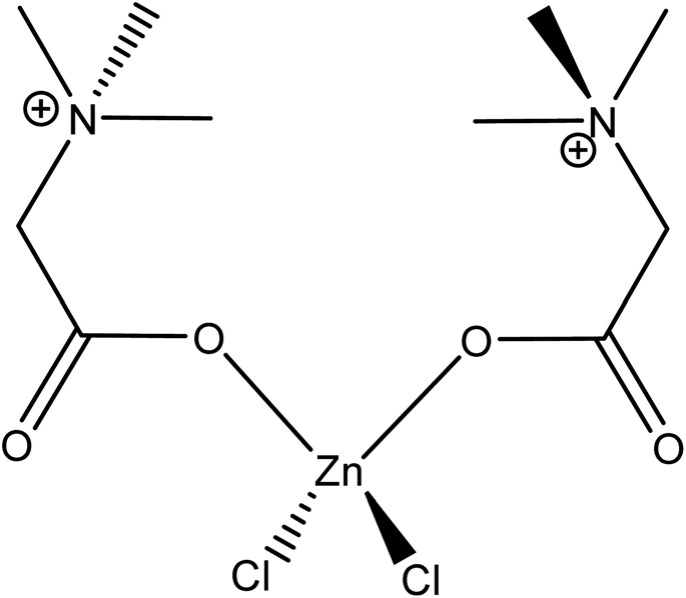
Presumed impurity Zn–betaine complex chemical formula [Zn(betaine)_2_Cl_2_].^48^

The first point to address is the appearance of Ostwald ripening, oriented attachment, and assembly behaviours in 40% betaine·HCl concentration at this extended time, whereas these phenomena were not apparent in the assessment at seven days. It can be speculated that the crystallisation precursor concentration remains higher (likely supersaturated) and lasts for longer in the case with 40% betaine·HCl, as compared to that with 10%. This higher, and longer lingering, concentration can be explained by the higher concentration of betaine·HCl resulting in more Zn-containing ions,^13,44^ which, in turn, provide a key reactant to forming a longer-lasting supply of the simonkolleite precursor, [Zn(betaine)_2_Cl_2_] (Fig. 11).

Thus, in the high concentration of betaine·HCl case (40%), the high supersaturation of precursors is the driving force for crystallisation during the initial period, from 2 to 7 days, leading to the critical crystal size remaining at a low level. As such, non-classical behaviours (namely Ostwald ripening) are not preferential during this period. From a thermodynamic standpoint, the surface energy of smaller crystal nuclei is always greater than that of a large crystal; therefore, the growth velocity of crystals is inversely proportional to size (*i*.*e*. tiny crystals have a higher rate of growth than large crystals). Newly produced tiny crystals can always catch up in size to larger crystals growing more slowly; overall, this results in narrowing of the crystal size distribution with time (‘size-distribution focusing’).^57^ However, this classical growth process is limited when the chain is broken, *i*.*e*. inadequate precursor concentration (*e*.*g*. zinc ions are not available). In this scenario, the supersaturation in the liquid phase then decreases (including dropping below supersaturation levels), resulting in Ostwald ripening overtaking as the dominant crystal growth mechanism. Of course, in cases with 10% betaine·HCl solutions the first period, with classical nucleation exists only briefly, if at all.

A second, competitive, behaviour of betaine·HCl molecules may also be in play, which must be considered, as it has the potential to act as a type of surfactant and, therefore, impact the crystal growth. The tail carboxyl group of betaine·HCl molecules releases hydrogen and leaves the carboxylate anion after the oxidation of metal zinc. The result is the formation of the zwitterion, [betaine]-, which can complex with zinc ions present on the polar face of the crystal. This would make it, effectively, an end-capping agent and inhibit growth along the *c*-axis direction (the [0,0,1] direction). As a result of this inhibition, this would promote the formation of sheet-like structures^58^ and encourage thinner structures. Herein, the particles formed at 40% betaine·HCl do appear thinner than those made in 10%. Additionally, there is significant evidence of other competing behaviours still occurring. Namely, signs of Ostwald ripening are apparent where tiny crystals in the solution can orientate and attach to large flakes to form a new layer. Monomers can stick to the raised edges of a new layer to further develop the layer. It allows simonkolleite to grow in the [0,0,1] direction and eventually form a thick hexagonal multilayer structure.

#### Reaction temperature

**Fig. 12:**
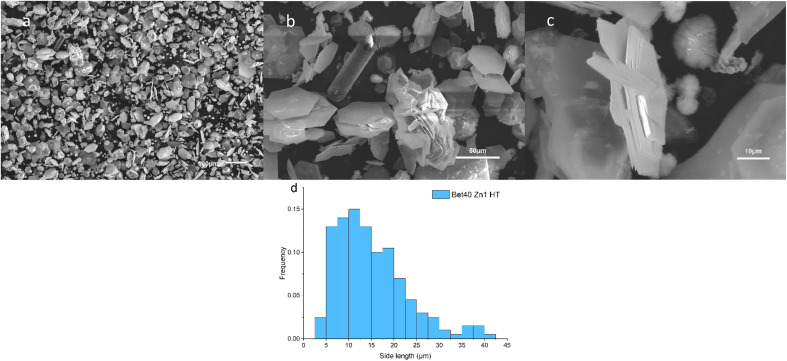
SEM images (a–c) of simonkolleite synthesized in 40% of betaine at 160 °C, 24 hours. A distribution of the side length for hexagonally-shaped simonkolleite particles is shown (d), where a step size of 2 μm is used in this instance.

A comparison of this synthesis was conducted at 160 °C for 24 h for a 40% betaine·HCl solution with Zn (Fig. 12). Unlike the lower temperature synthesis, various morphologies are observed, including hexagonal flakes, rods, plush balls, and lamellar clusters. At this elevated temperature, hexagonal platelets' side lengths observed appear to vary from 2.5 to 42.5 μm with a single crystal thickness of *ca*. 1 μm. This evidences that growth along the [0,0,1] direction (*c*-axis) is still inhibited at high temperatures. The particles formed flatter structures, resulting in a higher aspect ratio. The edges of plate crystals with defects are more clearly discernible. It may be inferred that individual hexagonal flakes are grown from individual nuclei. It may mean that the growth ability of individual nuclei at high temperatures is enhanced. Some complex stacking clusters were also observed in this system. In addition to face-to-face stacking, side-to-face, and side-to-side, even agglomerated petal-like structures occur. Therefore, it is believed that the morphological control of simonkolleite at high temperatures is weak, but it is capable of producing more complex assembled structures.

### Simonkolleite calcination

**Fig. 13:**
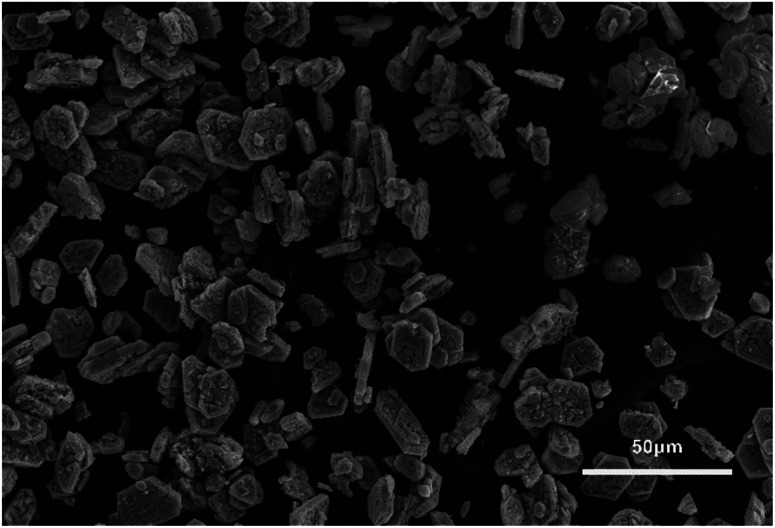
An SEM image of ZnO (confirmed by XRD) taken after the calcination (oxygen-free to 900 °C) of simonkolleite, which itself was formed in 10% of betaine for 15 days.

**Fig. 14:**
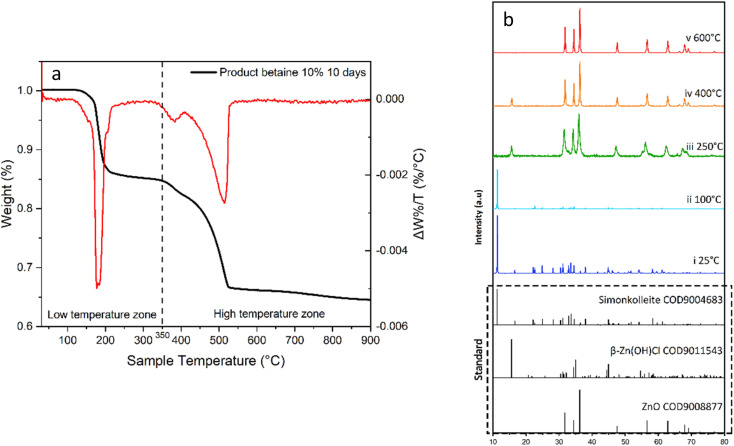
(a) The TGA analysis for product simonkolleite. The black curve stands for the residual mass of substance with increasing temperature; the red curve stands for the mass-loss rates at different temperatures. The original product was synthesized by 10% of betaine·HCl react with Zn for 10 days. (b) XRD data of product powder after calcination to different temperatures (100–600 °C) is presented on a sample synthesized in 10% betaine for 10 days: (i) blue curve is before calcination at 25 °C; (ii) light blue curve – calcined to 100 °C; (iii) green curve – calcined to 250 °C; (iv) orange curve – calcined to 400 °C; (v) red curve – calcined to 600 °C; spectra in dashed boxes represent standard diffraction peaks of simonkolleite, β-Zn(OH)Cl, and ZnO (crystallography open database).

The simonkolleite product was calcined (oxygen-free); SEM imaging was used to demonstrate whether the structural characteristics (*i*.*e*. hexagonal shape) were maintained (Fig. 13). The calcination was monitored by thermogravimetric analysis to 900 °C (Fig. 14a). This sample was subsequently characterised by XRD to confirm the conversion to ZnO and. To further establish the decomposition pathway, five individual calcination experiments were conducted to different temperatures (25, 100, 250, 400, and 600 °C) and characterised by XRD (Fig. 14b) on simonkolleite made with 10% of betaine·HCl for ten days.

#### Impact on morphology from calcination

The calcined product still maintains a hexagonal flake structure, although breakage and cracks are visible (Fig. 13). These new fractures in the crystal are likely due to the release of gaseous phases (including water and HCl) during the heating-induced decomposition of simonkolleite.^40^ In the crystal structure of simonkolleite (Fig. 1) the tetrahedral and octahedral structures, composed of coordinated Zn–O–Cl atoms, have a staggered stacking formation to make the crystal cell.^59^ During the thermal decomposition process, both H_2_O and HCl molecules leave the lattice (discussed subsequently). The phase transformation from simonkolleite to ZnO creates stress on the crystal lattice. Thus, the calcined ZnO exposes loosened porous structures at the nanoscale,^60^ but overall it retains the hexagonal frame formed in the synthesis of simonkolleite. It indicates that adopting simonkolleite as a calcined precursor has the potential to prepare hexagonal sheet-like ZnO.

#### Decomposition process

The thermal decomposition (Fig. 14) process can be roughly divided into two regions, the low-temperature zone (<350 °C) and the high-temperature zone (>350 °C). In the low-temperature zone, the TGA curve indicates that the product was very dry (*i*.*e*. no measurable free water present) because the product's weight remains unchanged until well after 100 °C. The XRD spectrum also shows little phase change from 25 to 100 °C (Fig. 14b, i. blue and ii. light blue).

Three key thermal decomposition reactions of simonkolleite have been identified at <350 °C. The XRD characterisation of the product calcined at 250 °C reveals the Zn(OH)Cl and ZnO (Fig. 14b, iii. green); there is no evidence of simonkolleite. The simonkolleite first decomposes into zinc oxide and 2β-Zn(OH)Cl at 170 °C (water is also released); if this reaction it goes to completion, a mass loss of 13.05% is expected (eqn (1)^61^).

1 Zn_5_(OH)_8_Cl_2_·H_2_O → 3ZnO + 2β-Zn(OH)C**l + 4H**_**2**_**O (g)**

Subsequently, 2β-Zn(OH)Cl is further decomposed into zinc oxide (ZnO), hydrated zinc chloride (ZnCl_2_·0.25H_2_O), and water at 220 °C (eqn (2)). The water-derived mass loss is 2.44% theoretically, with reaction completion.

2 2β-Zn(OH)Cl → ZnO + ZnCl_2_·0.25H_2_O + **0.75H**_**2**_**O(g)**

Subsequently, ZnCl_2_·0.25H_2_O is subject to decomposition itself, releasing HCl >230 °C. The remaining zinc is converted into ZnCl_2_ and ZnO, respectively (eqn (3)).

3 ZnCl_2_·0.25H_2_O → 0.25ZnO + **0.5HCl(g)** + 0.75ZnCl_2_

Consider the following cases, where all low-temperature zone reactions occur eqn (1)–(3) to completion. This results in a theoretical cumulative overall mass loss of 18.8%. Experimentally, the mass loss at 250 °C is 16.1%, presenting quite good agreement with the calculated expected loss and simultaneously indicating that all three reactions do not go to completion. The latter statement is consistent with the XRD results where no simonkolleite remains (*i*.*e*. eqn (1) goes to completion), but evidence of 2β-Zn(OH)Cl is apparent (*i*.*e*. eqn (2) does not go to completion).

Complex decomposition reactions can occur in the high-temperature zone (*i*.*e*. above 350 °C). Herein, a significant mass drop is observed in this high-temperature zone reaching an eventual residual mass *ca*. 67% (of the original mass). The mass loss predominantly occurs between 350 °C to 525 °C, with a small continuing decline after 525 °C. At a calcination temperature of 400 °C, the composition of the calcined product remains 2β-Zn(OH)Cl and ZnO. Notably, a characteristic peak of 2β-Zn(OH)Cl (2*θ* = 15.603°, [0,0,−2]) is decreasing (Fig. 14b, iv orange) relative to the spectrum at 250 °C. Simultaneously, the intensity of ZnO peaks (2*θ* = 31.773°, [−1,0,0]; 34.420°, [0,0,−2]; 36.256°, [−1,0,−1]) is increasing relatively speaking. When the temperature is increased to 600 °C, there is no longer evidence of 2β-Zn(OH)Cl and only peaks attributable to ZnO remain (Fig. 14b, v. red).

**Fig. 15:**
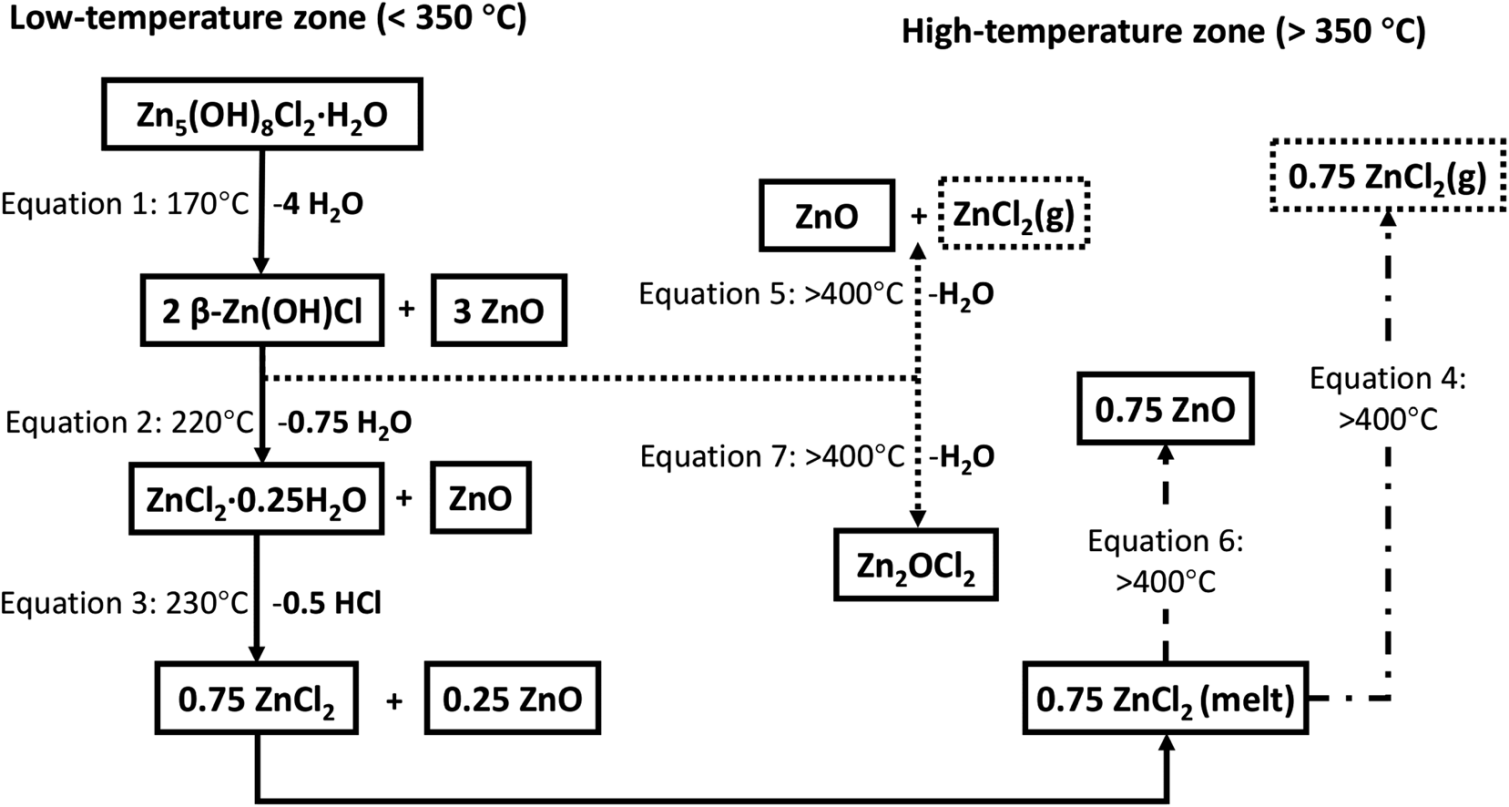
Schematic of a series of reaction equations for the decomposition process of simonkolleite; the residual substances are represented in the solid box, and volatile substances are represented by the dashed box. The arrows represent the reaction direction of the thermal decomposition process, and the solid arrows represent the most likely reactions.^45,62,63^

In the higher temperature region the removal of ZnCl_2_, *via* either eqn (4) (direct vaporisation from melt state) or eqn (6), occurs. The remaining 2β-Zn(OH)Cl, which was not converted in the low temperature range, can degrade in the high temperature region *via* two alternative pathways (eqn (5) or (7)) (Fig. 15).

Several potential contributing mechanisms can be considered in this high-temperature conversion. Whilst various mechanisms are possible, it is critical to consider that ZnCl_2_ is highly hygroscopic and does not tend to melt and evaporate in the presence of water. Anhydrous ZnCl_2_ is thermodynamically unstable and readily absorbs water from the environment to form hydrates. Therefore, the moisture of calcining environment impacts the decomposition process.^64^ Jones' research^63^ suggests ZnCl_2_ can melt at 320 °C and may vaporise at >400 °C (eqn (4)).

4 0.75ZnCl_2_(melt) ⇄ **0.75ZnCl**_**2**_**(g)**

A kinetically dominated reaction releasing water and ZnCl_2_ (eqn (5)) can take 2β-Zn(OH)Cl reactant and form ZnO. The theoretical volatilisation temperature of ZnCl_2_ is 400 °C, potentially contributing to the significant mass loss observed between 400 and 535 °C.

5 2β-Zn(OH)Cl → ZnO + **ZnCl**_**2**_**(g) + H**_**2**_**O(g)**

Considering the hygroscopic nature of ZnCl_2_, an alternative decomposition route of the hydrate, ZnCl_2_·0.25H_2_O, likely contributes to the mass losses observed. The result of this pathway are losses from gaseous HCl and H_2_O along with solid ZnO (eqn (6)). A second alternative pathway for the decomposition of 2β-Zn(OH)Cl has been suggested (eqn (7)).^62,65^

6 0.75ZnCl_2_ + 1.5H_2_O(g) → 0.75 [ZnCl_2_·2H_2_O] → 0.75 ZnO + **1.5HCl(g) + 0.75H**_**2**_**O(g)**

7 2β-Zn(OH)Cl → Zn_2_OCl_2_ + **H**_**2**_**O(g)**

**Fig. 16:**
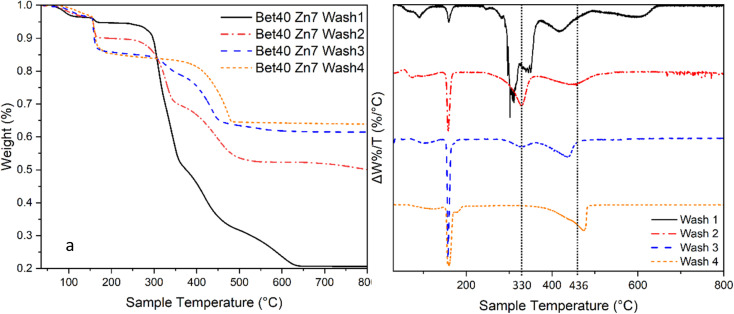
In this two part figure, illustrating the same data the TGA curve is shown on the left and the first derivative of mass loss plot with different washing times is shown on the right.

The onset of mass loss at 350 °C suggests that there may be either (1) the humidity in the calciner reducing the volatilisation temperature of ZnCl_2_ or (2) trapped volatile impurities released in this temperature range (Fig. 16). There is significant evidence that the latter explanation is the cause of this apparent early onset of mass loss. When varying degrees of washing were implemented, there was a significant impact on the losses measured in the range of 330–400 °C. Samples washed fewer times, *i.e.* higher retention of Zn–betaine complexes in particular, were observed. It is suggested that it is the Zn–betaine complex^48,66^ (Fig. 11), rather than pure betaine·HCl, because its decomposition temperature (330 °C) is significantly higher than betaine·HCl itself (*ca*. 250 °C).^67,68^ As can be seen, the peak at *ca*. 330 °C is notable because it is virtually eliminated with adequate washing. Therefore, this loss contributes *ca*. 3% of the total mass loss, leaving 11.2% attributable to the high decomposition reactions of 2β-Zn(OH)Cl and ZnCl_2_.

## Conclusion

In this work, hexagonal flakes of simonkolleite (Zn_5_(OH)_8_Cl_2_·H_2_O) have been successfully synthesised in a betaine·HCl solution resulting in the direct oxidation of ZnO (oxidative ionothermal synthesis). The product was characterised by XRD and TGA, where high purities of simonkolleite were reached. The size of the simonkolleite crystals, including thickness and diameter, can be adjusted by betaine·HCl concentration, reaction time, and reaction temperature.

The concentration of betaine·HCl is a dominant parameter in crystal size; overall, higher betaine·HCl concentration tested (40%) resulted in smaller and thinner simonkolleite flakes than the lower betaine·HCl (10%). Simonkolleite formed at 40% results in crystals preferentially forming individual flakes and do not show a significant tendency towards aggregation or other coalescence behaviours. By contrast, simonkolleite formed with 10% betaine solution showed distinctly different behaviours with the appearance of Ostwald ripening and attachment growth. The result, in these simonkolleite crystals, is that they appear as aggregates rather than as individual crystals. At a longer time for reaction, 15 days, the difference distribution in crystal size was quite notable for the different reaction conditions. The elevated reaction temperature tested showed a broader range of crystals morphologies, by contrast to the tests conducted at 40 °C, which consistently only show hexagonal flake structures. In addition, the calcination of simonkolleite to ZnO retains the hexagonal structure on the micron scale. This investigative work highlights how the production of hexagonally-shaped simonkolleite, or ZnO, can be obtained using an environmentally benign solution. Under suitable conditions, the crystal size can be controlled to be relatively uniform, and the distribution is within 0.5 microns. Moreover, control of the simonkolleite morphology and understanding of the chemistry of formation provide underpinning knowledge to exploit this method for production.

## Conflicts of interest

There are no conflicts to declare.

## Supplementary Material
